# Non-Specific Gastric Inflammation in Children is Associated with Proton Pump Inhibitor Treatment for More than 6 Weeks

**DOI:** 10.3389/fped.2014.00003

**Published:** 2014-01-20

**Authors:** Eduardo Rosas-Blum, Nina Tatevian, Syed Shahrukh Hashmi, Jon Marc Rhoads, Fernando Navarro

**Affiliations:** ^1^Division of Pediatric Gastroenterology, Department of Pediatrics, Texas Tech University Health Sciences Center, El Paso, TX, USA; ^2^Department of Pathology and Laboratory Medicine, University of Texas Health Science Center, Houston, TX, USA; ^3^Division of Pediatric Gastroenterology, Department of Pediatrics, University of Texas Health Science Center, Houston, TX, USA; ^4^Division of Pediatric Gastroenterology, Department of Pediatrics, Children’s Memorial Hermann Hospital, Houston, TX, USA

**Keywords:** pediatrics, gastric biopsies, gastric inflammation, duodenal inflammation

## Abstract

**Background and Aims:** Non-specific gastric inflammation (NSGI) is a commonly reported pathological finding. We investigated if it is associated with the use of proton pump inhibitors (PPIs) in children at a single tertiary center.

**Methods:** We performed an IRB-approved chart review of all endoscopy and biopsy reports of patients who underwent esophagogastroduodenoscopy between July 2009 and July 2010 (*n* = 310). Demographic data, dose, duration of exposure to PPI, and biopsy results were collected and analyzed. All esophageal, gastric, and duodenal biopsies were independently reviewed by a pathologist. Patients with acute gastritis, moderate/severe chronic gastric inflammation, or *Helicobacter pylori* infection were excluded. The presence of NSGI was compared between patients exposed and not exposed to PPI as well as between patients with different doses and durations of PPI exposure to assess for potential associations.

**Results:** A total of 193 patients were included: 88 (46%) had a history of PPI use and 48 (25%) were found to have NSGI. Compared to patients not exposed to PPI, the odds ratio of NSGI in patients exposed to PPIs was 2.81 (95% CI: 1.36–5.93). The odds ratio of NSGI in patients exposed to PPI for >3 months was 4.53 (95% CI: 1.69–11.97). Gender, ethnicity, and age were not associated with NSGI. No histological differences were found in the esophagus and duodenum between patients exposed and not exposed to PPI.

**Conclusion:** This study found that PPI exposure is associated with NSGI with a higher risk for those exposed for >3 months. As the clinical implications of NSGI are not known, judicious use of PPIs is needed. Prospective studies are required to confirm and to determine the etiologic factors (i.e., alteration of the gastric pH, serum gastrin) that may be related with the presence of NGSI.

## Introduction

Although proton pump inhibitors (PPIs) have shown a remarkable tolerability profile, the clinical use of these agents in children is based on adult data (1). In recent years, several studies have shown that PPIs are associated with an increased risk of infectious complications (pneumonia, *Clostridium difficile* infection, small bowel bacterial overgrowth) and nutritional deficiencies (vitamin B_12_ deficiency, osteoporosis) (2–4). Also, elevation of the gastric pH related to PPI usage may have deleterious effects on the gastrointestinal tract, including delayed gastric emptying, increased intestinal bacterial translocation, decreased gastric mucus viscosity, changes in the normal microbial flora, and possible impaired neutrophil function *in vitro* (5). Despite their safety profile, there are concerns regarding the long-term use of these agents (3, 6, 7).

Elevated serum gastrin level is a reproducible finding in patients taking PPIs, but its true clinical significance is yet unknown (2, 5, 8). As gastrin is a potent trophic hormone in the stomach (6), hypergastrinemia has been associated with changes in gastric histology in patients after long-term PPI use. These changes include gastric polyps, gastric nodules, and parietal cell hyperplasia (8–10). Several studies have reported non-specific gastric inflammation (NSGI) as one of the most common histological findings (11, 12) independent of PPI use. NSGI is characterized by mild chronic inflammation with focal collections of lymphocytes and plasma cells in the lamina propria predominantly in the antrum, and for which there is no identified cause (12). Mild chronic inflammation was defined according to the Sydney classification for gastritis (13, 14). The presence of tissue eosinophilia with peak eosinophil count was also recorded for all biopsies. The diagnosis of NSGI was given to those biopsies with focal collections of lymphocytes and plasma cells in the lamina propria.

In our practice, there has been a noticeable increase in the number of patients with this pathologic finding in recent years.

The primary aim of this study was to determine if NSGI is associated with PPI exposure. The secondary aims included the description of morphological changes in the esophagus and the duodenum (i.e., eosinophilic infiltration, peak eosinophil count), as well as to determine if there is an association between PPI duration, dose, and these changes.

## Materials and Methods

After obtaining approval from the Institutional Review Board, at the University of Texas Health Science Center, we performed a retrospective review of the medical records of all children who underwent esophagogastroduodenoscopy (EGD) from July 2009 to July 2010 at Children’s Memorial Hermann Hospital (a university affiliated hospital). The following demographic data was ascertained from the medical records: age, sex, ethnicity, and BMI. Endoscopy reports, procedure indication, and the associated pathology reports were reviewed. Dose, duration, and type of PPI used prior to endoscopy were obtained. Patients up to 18 years of age, who had a normal EGD and had gastrointestinal biopsies taken were included. Patients with history of *Helicobacter pylori* infection, peptic ulcer disease, celiac disease, eosinophilic gastroenteritis, reflux esophagitis, eosinophilic esophagitis, acute gastritis, or moderate/severe chronic inflammation of the stomach were excluded.

Subjects were divided in two groups for analysis: one group with patients who had PPI exposure and the other group with patients with no exposure to PPI prior to the EGD. Subjects with inconsistent PPI use were included if their PPI exposure was for at least six consecutive weeks prior to the endoscopy.

### Histological analysis

All biopsies were reviewed by a single pathologist who was blinded to the patient’s clinical information. Esophageal, gastric, and duodenal biopsies were evaluated for adequacy of the submitted tissue, architectural changes, inflammation, peak eosinophil count, and presence of *H. pylori*. The presence of tissue eosinophilia with peak eosinophil count was also recorded for all biopsies.

### Statistical analysis

Descriptive statistics was used for demographics, endoscopy indications, PPI usage pattern, and biopsy results. Odds ratios and 95% confidence intervals were calculated to evaluate any difference in odds of PPI use among individuals with and without gastric inflammation. Stratified analysis and multivariable logistic regression models were used to adjust PPI type, duration (≤1.5, 1.5, 1.5–3.0, and >3 months), dosage (≤0.5, 0.5–1.0, and >1.0 mg/kg/day), gender, ethnicity, age, and obesity. Statistical significance was assumed at *p* < 0.05 or a confidence interval that did not include the null.

## Results

**Figure 1:**
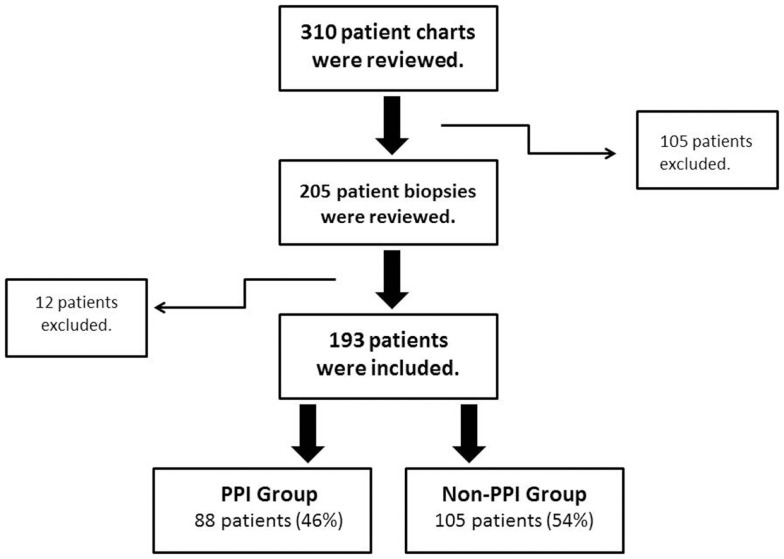
**Patient inclusion and exclusion algorithm**. Flow chart of the inclusion/exclusion criteria. PPI, proton pump inhibitors.

After the initial review of 310 charts, 117 subjects were excluded (reasons for their exclusion are listed in Table 1) and 193 children were included; 88 (45.6%) had PPI exposure prior to EGD (Figure 1). There were no significant differences between PPI-exposed and non-exposed on the basis of gender, age, ethnicity, and BMI (Table 2).

**Table 1:** **Patients excluded from the analysis**.

Initial review
*H. pylori* gastritis	28
Eosinophilic esophagitis	17
Inflammatory bowel disease	10^a^
Celiac disease	8
Peptic ulcer	7
Eosinophilic gastroenteritis	6
No gastric biopsies	6
Other	23^b^
After histological analysis
No biopsies available	6
Colitis	2
Acute duodenitis	1
Esophageal ulcer	1
Eosinophilic esophagitis	1
*H. pylori* gastritis	1

*^a^Crohn’s disease (7), ulcerative colitis (3)*.

*^b^Other: lupus, history of liver transplant, vasculitis, esophageal ulcers, short bowel syndrome, pancreatitis, *Candida* esophagitis, lymphoma, achalasia, renal failure, esophageal strictures, and immunodeficiency*.

**Table 2:** **Patient demographics**.

	PPI use	No PPI use	*p*-Value
Total, *n* (%)	88 (45.6)	105 (54.4)	
<6 weeks	33 (37.5)	
>6 weeks	43 (48.9)	
Intermittent	12 (13.6)	
Gender, *n* (%)
Female	41 (21.2)	55 (28.5)	*p* = 0.423
Male	47 (24.4)	50 (25.9)	
Age, mean (std. dev.)	9.35 (5.09)	8.27 (5.63)	*p* = 0.171
Ethnicity, *n* (%)^a^
White	46 (52.3)	41 (39.4)	*p* = 0.216
Hispanic	23 (26.1)	40 (38.5)	
African American	12 (13.6)	12 (11.5)	
Other	7 (8.0)	11 (10.6)	
BMI, median (range)^b^	19.11 (13.1–32.17)	18.15 (12.69–41.8)	*p* = 0.456
*Z* score, median (IQR)	0.46 (−3.2–4.65)	0.13 (−4.27–3.67)	*p* = 0.231

*^a^Data missing for one subject with no history of PPI use*.

*^b^Data missing for 6 subjects with history of PPI use and 13 subjects with no history*.

### PPI therapy

Lansoprazole was used by 61 subjects (69.3%), esomeprazole by 11 subjects (12.4%), other PPIs (omeprazole, rabeprazole, or pantoprazole) by 9 subjects (10.2%). More than one type of PPI was used by five (5.7%) subjects during the treatment course. The median daily PPI dose was 0.89 mg/kg (IQR 0.53–1.20 mg/kg). Among the patients with PPI exposure, 24 subjects (27.3%) received PPI for <1 month, 14 subjects (15.9%) between 1 and 2 months, 38 subjects (43.2%) for >2 months, and 12 subjects (13.6%) used PPI inconsistently for at least 6 weeks.

### Histological findings

**Figure 2:**
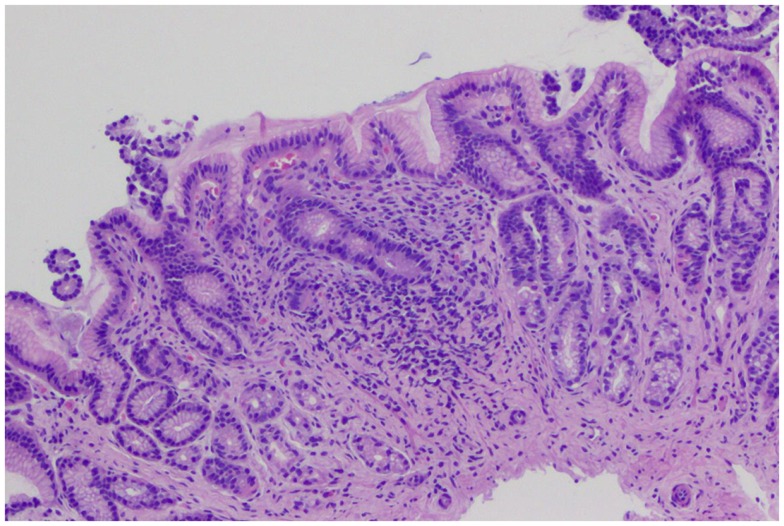
**Gastric biopsies with non-specific gastric inflammation**. Gastric antral-type mucosa at low power with lymphoplasmacytic inflammation expanding the lamina propria. The general architecture is preserved with no erosions or acute inflammation.

The biopsies of 193 patients were reviewed: 48 (24.8%) had NSGI (Figure 2); 7 (3.6%) subjects had NSGI with isolated lymphoid aggregate (ILA); 8 (4.1%) had NSGI with lymphoid follicle (LF) in the same biopsy specimen. Other gastric findings included: 27 patients (13.9%) had LFs and germinal centers in the absence of *H. pylori* infection; 11 (5.7%) had ILAs, represented by a lymphoid conglomerate without germinal center; and 3 (1.6%) had foveolar hyperplasia. The remaining 145 subjects (76%) had normal gastric histology. Gender, ethnicity, age, and BMI were not associated with NSGI in multivariable models.

Other histological findings included: 20 patients (10.4%) with mild duodenal chronic inflammation and 24 (12.4%) with a mild esophageal lymphocytic infiltrate.

### Stomach inflammation and PPI use

Among the patients with NSGI, 31/48 (64.6%) had PPI exposure and when compared to patients not exposed to PPI, their odds ratio of having NSGI was 2.81 (95% CI: 1.36–5.93). The odds ratio of NSGI for patients exposed to lansoprazole was 2.04 (95% CI 1.04–4.02) (the OR for patients exposed to other PPIs did not reach statistical significance).

In order to assess the association between duration of exposure to PPI and NSGI, PPI-exposed patients were stratified in two groups: <6 weeks of treatment and ≥6 weeks of treatment (Table 3). Patients with NSGI were four times more likely to have PPI exposure for >6 weeks when compared to patients without NSGI (OR 4.10, 95% CI 1.85–9.07). The odds were not increased if PPI exposure was briefer than 6 weeks. This higher risk of NSGI with increasing duration of PPI exposure was also found after further stratification into two additional groups: PPI exposure between 6 weeks and 3 months (OR 3.24, 95% CI 0.97–11.09) and PPI exposure for more than 3 months (OR 4.53, 95% CI 1.87–10.98).

**Table 3:** **Proton pump inhibitor duration dose category counts and OR (with 95% CI)**.

	NSGI^a^		Odds ratio^b^	95% Confidence interval
	
	Yes	No	
No PPI use	17	88	–	
PPI use	31	57	2.82	1.42–5.55
Duration <6 weeks	8	25	1.66	0.64–4.29
Dose 0.8–1 mg/kg/day	3	8	1.94	0.47–8.07
Dose >1 mg/kg/day	2	11	0.94	0.19–4.63
Duration ≥6 weeks	19	24	4.10	1.85–9.07
Dose 0.8–1 mg/kg/day	12	13	4.78	1.86–12.24
Dose >1 mg/kg/day	3	10	1.55	0.39–6.24

*^a^Numbers in subgroups do not add up to sums in parent groups due to missing data for duration (*n* = 12) and dose (*n* = 23)*.

*^b^All comparisons are to the non-PPI users*.

Also aimed to assess the association between dose of PPI and NSGI, patients exposed to PPI were compared to patients non-exposed to PPI by using two different PPI doses at the time of endoscopy. There was a statistically significant increased risk of NSGI with the standard PPI dose (0.8–1 mg/kg; *n* = 38) (OR 3.77, 95% CI 1.65–8.61); however, the risk was not statistically significant for higher doses (>1 mg/kg; *n* = 27) (OR 1.18, 95% CI 0.39–3.54) (Table 3). Additionally, a comparison of point estimates between PPI exposed patients with >6 weeks duration of treatment and those with <6 weeks duration, demonstrated higher odds ratios for each dose range among patients exposed to PPI for >6 weeks.

Although age and gender were not independently associated with either NSGI or PPI use, it was noted that the proportion of younger subjects (<13 years-preteen) was mostly of female gender (79.2% female vs. 60.8% male; *p* = 0.005). Gender appeared to be associated with PPI-associated NSGI, as females with NSGI had a greater than fivefold increased odds of having been exposed to PPI (OR 5.78, 95% CI 2.02–16.55) compared to females without NSGI; this association was not found in males (OR 1.50, 95% CI 0.60–3.76). After adjusting for age (preteen vs. teenage) this increased risk persisted for teenage females with a wide confidence interval given the size of this group of patients. It was also noted that age did not significantly influence the association between PPI exposure and NSGI in males.

### Duodenal and esophageal inflammation and PPI use

Among patients with mild chronic duodenal inflammation, compared with children without inflammation, the odds of PPI exposure were not significantly increased (OR 2.42, 95% CI 0.92–6.38). However, an increased risk of mild chronic duodenal inflammation was found with a PPI exposure of >6 weeks (OR 3.2, 95% CI 1.08–9.47) compared to children without inflammation. This risk was not increased for PPI exposure of <6 weeks (OR 0.90, 95% CI 0.18–4.58). No increased risk of mild chronic duodenal inflammation with different PPI doses in both groups was found. There was no association between PPI exposure, dose or duration of treatment, and esophageal lymphocytic inflammation.

### Eosinophil count and PPI use

The peak eosinophil count per single high power field (eos/hpf) was almost identical between those exposed and non-exposed to PPI. Patients with NSGI had a median count of 3.5 eos/hpf (range 1–12 eos/hpf), and those without NSGI had a median count of 2 eos/hpf (range 1–11 eos/hpf). Duration or dose of PPI treatment was not associated with changes in eosinophil count.

## Discussion

Our study shows an association between NSGI and long term exposure to PPIs. Patients who were exposed to PPI for more than 6 weeks had a nearly fourfold increased risk of NSGI. This association was even stronger in those who had PPI exposure for more than 3 months. In a separate analysis of the different types of PPI used, lansoprazole (66%), the most common PPI in this cohort had the highest risk. Additional studies need to be conducted to determine if specific PPIs are associated with a higher risk of NSGI.

The diagnosis of NSGI has been reported in prior retrospective studies. Pashankar et al. (11) reported that up to 57% of their cohort had a diagnosis of NSGI, but they did not mention if the patients had exposure to PPI. Tolia et al. (6) reported that NSGI was one of the most frequently observed histological findings in a pediatric cohort, being present in 15–38% of gastric biopsies. These patients were exposed to PPIs for at least 1 year (different PPIs had different risk of NSGI). In our study, NSGI was present in 24% of all gastric biopsies reviewed, and it was seen in 33% of patients who had been exposed to PPI before endoscopy. In addition, NSGI was seen in those patients who had been exposed to PPI for at least 6 weeks, providing a more specific and narrow time frame during which NSGI could develop. This change persisted as long as the PPI therapy was continued. Future studies will be needed to determine if NSGI resolves after PPI discontinuation and the length of time this would take.

Our study also showed that higher doses of PPI (>1 mg/kg) may not increase the risk of NSGI compared to the standard dose, but the likely explanation is because there were more patients receiving the standard dose (median PPI dose was 0.89 mg/kg/day in about half of our cohort). Another reason for this finding might be that clinicians were more attentive to patients receiving higher doses of PPI and may have limited the duration of treatment.

Favorable effectiveness and safety profiles have led to a significant increase in prescription of PPIs in the last decade (14–16). The “PPI trial” or short PPI use for 4–8 weeks is a common practice among clinicians as a diagnostic tool for gastroesophageal reflux disease (GERD) (17, 18) and to rule in “PPI-responsive eosinophilic esophagitis” (recent ACG guidelines). Many primary care practitioners and gastroenterologists continue PPI therapy for prolonged periods of time with no clear criteria for discontinuation (7, 19). Some patients could be receiving PPI therapy with no clear medical indication, perhaps as a placebo, or as means to maintain partial resolution of gastrointestinal symptoms. This practice was evident in our study as 43% of patients on PPI underwent an EGD for persistent symptoms despite the use of a PPI for more than 2 months. To our knowledge, there have been no studies exploring the role of prolonged acid suppression in patients who have partial resolution of symptoms (i.e., nausea, abdominal pain).

Although the clinical significance of NSGI has yet to be established, the results of this study support using these therapeutic agents for only the recommended trial period and at the standard dose. If patients fail the recommended therapeutic trial, practitioners should discontinue the medication and investigate other potential causes.

The authors of this study believe that because several studies have reported NSGI in children (a vulnerable population), investigations to understand the pathophysiology of NSGI are needed. For example, PPIs may be handled differently by different patients depending upon genetic differences. It is known that PPIs are metabolized by the hepatocyte CYP2C19 and CYP3A4 enzymes (1). Kearns et al. reported different genetic polymorphisms of CYP2C19 that can lead to differences in PPI pharmacokinetics (20). For instance, a patient with a poor metabolizer phenotype for CYP2C19 has a higher plasma concentration from a therapeutic dose of a PPI, which potentially could cause a more profound acid suppression, perhaps increasing the risk for NSGI.

In addition to the increased risk of NSGI in children receiving PPIs, gastric acid suppression can potentially interfere with the natural antibacterial protection of gastric acid (5, 21–23) as well as with the natural pH-dependent deactivation of refluxed pancreatic enzymes (24). The effect of prolonged gastric alkalinization on the risk of systemic and gastrointestinal infection also needs to be investigated. There have been many reports in adults and children of fundic gastric polyps and/or cysts associated to PPI use (5, 8). None of the EGD reports reviewed during this study reported these lesions, even in patients exposed to PPI for more than a year.

Although we acknowledge that the retrospective design of this study is its main limitation, we also recognize that its sample size is one of its strengths. Some of the other limitations of our study include the assumption that patients were taking the medication at the dose and for the duration as documented in their chart, and the assumption that gastric inflammation was not present before the patient was exposed to PPI. A prospective pediatric study to determine if PPI use increases the risk for NSGI would be unethical as patients would be required to have multiple endoscopies performed at different durations during their PPI treatment.

In conclusion, PPI exposure for more than 6 weeks was associated with NSGI, with the highest risk among patients using higher doses for longer periods of time. Although our study does not establish causality, in patients with persistent symptoms despite acid suppression, we question the benefit of continuing PPI therapy beyond this time. We hypothesize that the NSGI is related to a disruption in the normal gastric pH caused by PPIs use in genetically susceptible individuals. Future prospective studies are required for the understanding of the pathophysiology and natural history of these changes in the gastric mucosa.

## Author Contributions

Eduardo Rosas-Blum: study concept and design; acquisition of data; interpretation of the data; drafting of the manuscript. Nina Tatevian: study concept and design; acquisition of data; drafting of the manuscript. Shahrukh Hashmi: statistical analysis and interpretation of the data; drafting of the manuscript. Marc Rhoads: study concept and design; drafting of the manuscript. Fernando Navarro: study concept and design; interpretation of the data; drafting of the manuscript; study supervision.

## Conflict of Interest Statement

The authors declare that the research was conducted in the absence of any commercial or financial relationships that could be construed as a potential conflict of interest.

## References

[1] Gibbons TE Gold BD The use of proton pump inhibitors in children: a comprehensive review. Paediatr Drugs (2003) 5(1):25–40 10.2165/00148581-200305010-00003 12513104

[2] Thomson AB Sauve MD Kassam N Kamitakahara H Safety of the long-term use of proton pump inhibitors. World J Gastroenterol (2010) 16(19):2323–30 10.3748/wjg.v16.i19.2323 20480516 PMC2874135

[3] Ali T Roberts DN Tierney WM Long-term safety concerns with proton pump inhibitors. Am J Med (2009) 122(10):896–903 10.1016/j.amjmed.2009.04.014 19786155

[4] Nealis TB Howden CW Is there a dark side to long-term proton pump inhibitor therapy? Am J Ther (2008) 15(6):536–42 10.1097/MJT.0b013e31817149bf 19127138

[5] Lamberts R Brunner G Solcia E Effects of very long (up to 10 years) proton pump blockade on human gastric mucosa. Digestion (2001) 64(4):205–13 10.1159/000048863 11842276

[6] Tolia V Boyer K Long-term proton pump inhibitor use in children: a retrospective review of safety. Dig Dis Sci (2008) 53(2):385–93 10.1007/s10620-007-9880-7 17676398

[7] Heidelbaugh JJ Goldberg KL Inadomi JM Overutilization of proton pump inhibitors: a review of cost-effectiveness and risk [corrected]. Am J Gastroenterol (2009) 104(Suppl 2):S27–32 10.1038/ajg.2009.49 19262544

[8] Drut R Altamirano E Cueto Rua E Omeprazole-associated changes in the gastric mucosa of children. J Clin Pathol (2008) 61(6):754–6 10.1136/jcp.2007.052480 18077769

[9] Pashankar DS Israel DM Gastric polyps and nodules in children receiving long-term omeprazole therapy. J Pediatr Gastroenterol Nutr (2002) 35(5):658–62 10.1097/00005176-200211000-00013 12454582

[10] Pashankar DS Israel DM Jevon GP Buchan AM Effect of long-term omeprazole treatment on antral G and D cells in children. J Pediatr Gastroenterol Nutr (2001) 33(5):537–42 10.1097/00005176-200111000-00005 11740225

[11] Pashankar DS Bishop WP Mitros FA Chemical gastropathy: a distinct histopathologic entity in children. J Pediatr Gastroenterol Nutr (2002) 35(5):653–7 10.1097/00005176-200211000-00012 12454581

[12] Dohil R Hassall E Jevon G Dimmick J Gastritis and gastropathy of childhood. J Pediatr Gastroenterol Nutr (1999) 29(4):378–94 10.1097/00005176-199910000-00004 10512396

[13] Price AB Misiewicz JJ Sydney classification for gastritis. Lancet (1991) 337(8734):174 10.1016/0140-6736(91)90836-E 1670809

[14] Dixon MF Genta RM Yardley JH Correa P Classification and grading of gastritis. The updated Sydney System. International Workshop on the Histopathology of Gastritis, Houston 1994. Am J Surg Pathol (1996) 20(10):1161–81 10.1097/00000478-199610000-00001 8827022

[15] Barron JJ Tan H Spalding J Bakst AW Singer J Proton pump inhibitor utilization patterns in infants. J Pediatr Gastroenterol Nutr (2007) 45(4):421–7 10.1097/MPG.0b013e31812e0149 18030207

[16] van der Pol RJ Smits MJ van Wijk MP Omari TI Tabbers MM Benninga MA Efficacy of proton-pump inhibitors in children with gastroesophageal reflux disease: a systematic review. Pediatrics (2011) 127(5):925–35 10.1542/peds.2010-2719 21464183

[17] Aanen MC Weusten BL Numans ME de Wit NJ Baron A Smout AJ Diagnostic value of the proton pump inhibitor test for gastro-oesophageal reflux disease in primary care. Aliment Pharmacol Ther (2006) 24(9):1377–84 10.1111/j.1365-2036.2006.03121.x 17059519

[18] Lee SH Jang BI Jeon SW Kwon JG Kim EY Cho KB A multicenter, randomized, comparative study to determine the appropriate dose of lansoprazole for use in the diagnostic test for gastroesophageal reflux disease. Gut Liver (2011) 5(3):302–7 10.5009/gnl.2011.5.3.302 21927658 PMC3166670

[19] Illueca M Wernersson B Henderson C Lundborg P Maintenance treatment with proton pump inhibitors for reflux esophagitis in pediatric patients: a systematic literature analysis. J Pediatr Gastroenterol Nutr (2010) 51(6):733–40 10.1097/MPG.0b013e3181e2acfd 20808247

[20] Kearns GL Winter HS Proton pump inhibitors in pediatrics: relevant pharmacokinetics and pharmacodynamics. J Pediatr Gastroenterol Nutr (2003) 37(Suppl 1):S52–9 10.1097/00005176-200311001-00011 14685079

[21] Cocanour CS Dial ED Lichtenberger LM Gonzalez EA Kozar RA Moore FA Gastric alkalinization after major trauma. J Trauma (2008) 64(3):681–7 10.1097/TA.0b013e3181641bdb 18332808

[22] Kato S Fujimura S Kimura K Nishio T Hamada S Minoura T Non-*Helicobacter* bacterial flora rarely develops in the gastric mucosal layer of children. Dig Dis Sci (2006) 51(4):641–6 10.1007/s10620-006-3185-0 16614982

[23] Sanduleanu S Jonkers D De Bruine A Hameeteman W Stockbrugger RW Non-*Helicobacter pylori* bacterial flora during acid-suppressive therapy: differential findings in gastric juice and gastric mucosa. Aliment Pharmacol Ther (2001) 15(3):379–88 10.1046/j.1365-2036.2001.00888.x 11207513

[24] Dial EJ Tran DM Romero JJ Zayat M Lichtenberger LM A direct role for secretory phospholipase A2 and lysophosphatidylcholine in the mediation of LPS-induced gastric injury. Shock (2010) 33(6):634–8 10.1097/SHK.0b013e3181cb9266 19940811 PMC2875268

